# Research progress on the association between viruses and cardiac diseases

**DOI:** 10.1128/jvi.00383-26

**Published:** 2026-06-09

**Authors:** He-Min Yu, Ming-Liang Zhu, Yan-Li Zhao, Jia-Xiang Tan, Shao-Chao Luo, Pan-Pan Pang, Chang-Bo Zheng

**Affiliations:** 1 School of Pharmaceutical Science and Yunnan Key Laboratory of Pharmacology for Natural Products, Kunming Medical University 71240, Kunming, Yunnan, China; 2 Yunnan Key Laboratory of Cross-Border Infectious Disease Control and Prevention and Novel Drug Development, Kunming Medical University, Kunming, Yunnan, China; 3 Yunnan Key Laboratory of Southern Medicinal Utilization, Yunnan University of Chinese Medicine, Kunming, Yunnan, China; Indiana University Bloomington, Bloomington, Indiana, USA

**Keywords:** viral infection, cardiac diseases, antiviral therapy, prevention and risk factor control

## Abstract

Virus-related cardiac diseases encompass a diverse spectrum of cardiovascular pathologies caused by direct viral infection and indirect immune-mediated injury. Major cardiotropic and cardioactive viruses—including severe acute respiratory syndrome coronavirus 2, influenza virus, human immunodeficiency virus, arboviruses (dengue virus, chikungunya virus, Zika virus), enteroviruses (coxsackievirus B3), and human cytomegalovirus—contribute to myocardial injury manifesting as myocarditis, pericarditis, arrhythmias, acute and chronic heart failure, and thromboembolic complications. Mechanisms of cardiac involvement are multifactorial, involving direct infection of cardiac cells leading to cytopathic effects and innate immune activation, dysregulated adaptive immune responses promoting myocardial inflammation and fibrosis, electrophysiological disturbances, and systemic effects such as endothelial dysfunction and prothrombotic states that precipitate ischemic events. Clinical manifestations range from subclinical injury to fulminant myocardial inflammation and may also include long-term sequelae, such as post-acute cardiovascular symptoms, as observed in long COVID cases. Therapeutic strategies emphasize early antiviral treatment when effective agents exist, judicious immunomodulation based on viral replication status, and supportive management of cardiac dysfunction and arrhythmias. Prevention through vaccination, personal protective measures, vector control, and optimization of cardiovascular risk factors remains pivotal to reduce the burden of virus-associated cardiovascular diseases. Future research requires improved diagnostic precision, mechanistic elucidation, and randomized trials to optimize antiviral and immunomodulatory interventions and to develop vaccines for currently unmet viral targets. An integrated, mechanism-informed approach across prevention, acute management, and long-term care is essential to mitigate the cardiovascular morbidity and mortality attributable to viral infections.

## INTRODUCTION

Viruses have existed long before human history and are ubiquitous in the human environment. Humans acquire viral infections through multiple routes, including the respiratory tract (e.g., inhalation of aerosols or droplets), the gastrointestinal tract (e.g., ingestion of contaminated food or water), contact transmission—either direct (person-to-person) or indirect (via contaminated objects or surfaces)—and vector-borne transmission via arthropods such as mosquitoes and ticks, which is particularly relevant for arboviruses. Importantly, only a subset of viruses exhibit clinically meaningful cardiac tropism or trigger host responses that culminate in cardiovascular injury. Nonetheless, viral infections can affect the cardiovascular system through direct and indirect mechanisms and are increasingly recognized as contributors to an elevated risk of major cardiovascular diseases (1). A substantial body of evidence supports a close association between viral infection and cardiac pathology. Viral illness may precipitate a spectrum of cardiovascular manifestations, including myocarditis, pericarditis, arrhythmias, and acute or chronic heart failure (2–5). Proposed mechanisms include direct injury to cardiomyocytes and other cardiac-resident cells, immune-mediated damage (e.g., exaggerated inflammation, cytokine-driven injury, and autoimmunity), and perturbations of cardiac electrophysiology that promote conduction abnormalities and malignant arrhythmias. In addition, systemic effects of infection—such as endothelial dysfunction, plaque destabilization, and a prothrombotic state—may precipitate acute ischemic events and cerebrovascular complications (6–8). Multiple viral pathogens have been implicated in major adverse cardiovascular events (MACEs). These include severe acute respiratory syndrome coronavirus 2 (SARS-CoV-2), influenza virus, HIV, arboviruses (dengue virus [DENV], chikungunya virus [CHIKV], Zika virus [ZIKV]), enteroviruses (coxsackievirus B3 [CVB3]), and human cytomegalovirus (HCMV). Reported MACEs encompass acute myocardial infarction, stroke, heart failure exacerbation or new-onset heart failure, clinically significant arrhythmias, and myocarditis (3, 9–14).

## VIRAL INFECTIONS ASSOCIATED WITH CARDIOVASCULAR DISEASES

### SARS-CoV-2

Since the initial reports in 2019, the coronavirus disease 2019 (COVID-19) pandemic, caused by SARS-CoV-2, has resulted in more than 7 million deaths worldwide (15). The most common proximate cause of death in severe cases is respiratory failure due to viral pneumonia and acute respiratory distress syndrome (16). However, cardiovascular complications—including acute heart failure, clinically significant arrhythmias, and thromboembolic events—also substantially contribute to morbidity and mortality (17).

SARS-CoV-2 entry into host cells is primarily mediated by binding of the viral spike protein to angiotensin-converting enzyme 2 (ACE2), followed by membrane fusion and/or receptor-mediated endocytosis (18). ACE2 is a membrane-bound carboxypeptidase that also serves as a functional viral receptor; it is highly expressed in several organs, including the lungs, kidneys, and heart, and soluble ACE2 can be detected in plasma (19, 20). SARS-CoV and SARS-CoV-2 share substantial sequence homology (approximately 76% amino acid identity), and ACE2 is a key determinant of host range and transmissibility for related coronaviruses (21–23). Within the heart, experimental studies indicate that SARS-CoV-2 shows relatively higher tropism for cardiomyocytes and pericytes than for macrophages, fibroblasts, or endothelial cells, although these findings may vary by model system and disease context (24, 25). Moreover, increased myocardial ACE2 expression reported in conditions such as heart failure and diabetes has been proposed as a potential factor influencing susceptibility and/or disease severity in patients with pre-existing cardiovascular risk (24, 26–29).

**Fig 1:**
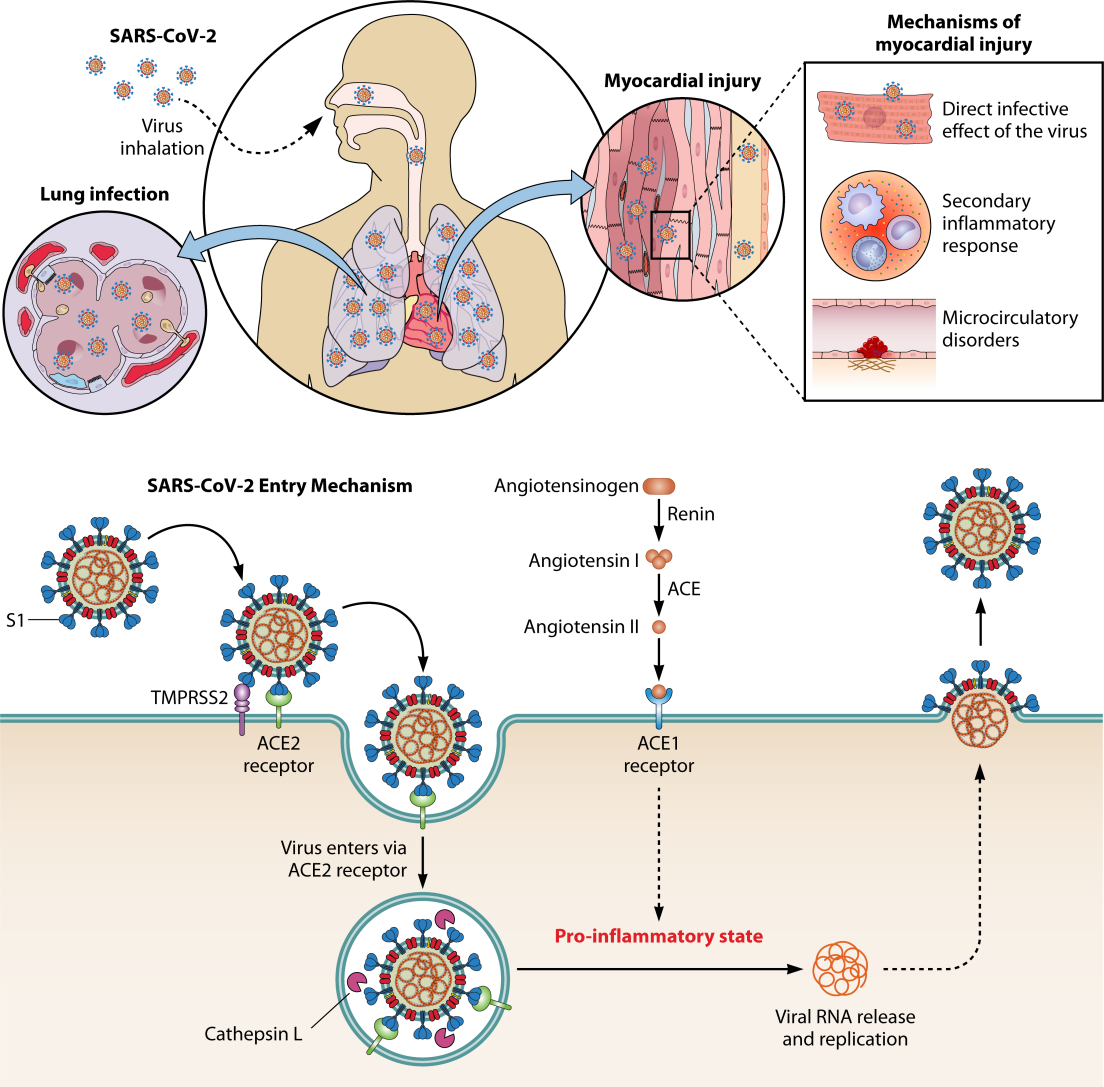
Mechanism of SARS-CoV-2-induced myocardial injury. SARS-CoV-2: severe acute respiratory syndrome coronavirus 2; ACE: angiotensin-converting enzyme; ACE2: angiotensin-converting enzyme 2; TMPRSS2: transmembrane serine protease 2.

Cardiac involvement in COVID-19 is clinically heterogeneous, encompassing myocarditis or myocarditis-like syndromes, pericarditis, arrhythmias, heart failure (including *de novo* dysfunction and exacerbation of established disease), and vascular complications such as coronary inflammation and thrombosis (30–33). Epidemiological studies have identified COVID-19 as a risk factor for acute myocardial infarction and ischemic stroke, consistent with these vascular clinical manifestations (34). Mechanistically, SARS-CoV-2–associated cardiac injury is likely multifactorial, reflecting a variable contribution from direct viral infection of cardiac cells, immune-mediated injury, microvascular dysfunction, and systemic inflammation with prothrombotic and hemodynamic consequences (35) (Fig. 1). Consistent with this, epidemiologic studies have demonstrated that biochemical and/or imaging evidence of myocardial injury is associated with worse outcomes and higher mortality in hospitalized patients (36). Substantial evidence from autopsy and tissue-based studies has demonstrated the presence of SARS-CoV-2 RNA, antigen, and related viral material in cardiovascular tissues, including myocardial tissue and coronary arteries (8, 37). However, these findings do not uniformly establish widespread productive cardiomyocyte infection in all cases, and viral RNA may be undetectable in some myocardial samples at the time of examination, likely reflecting heterogeneity in disease stage, tissue sampling, and viral persistence (38, 39). This observation may indicate that indirect immune or inflammatory mechanisms predominate in a substantial proportion of patients. However, alternative explanations must be considered, including that the virus had already been cleared by the host immune response prior to sampling, or that the focal nature of viral myocarditis combined with limited tissue sampling could lead to false-negative results. Collectively, these findings underscore that indirect mechanisms play a significant role in the pathogenesis of virus-associated cardiovascular injury while also highlighting the diagnostic challenges in confirming direct cardiac infection in human studies. Experimental models provide additional insight into potential direct effects on cardiomyocytes. In human pluripotent stem cell–derived cardiomyocytes, SARS-CoV-2 infection has been associated with impaired contractility, induction of inflammatory mediators, sarcomere disruption, and cell death, collectively compromising electrical and mechanical function (6, 40). In a mouse model with cardiomyocyte-restricted SARS-CoV-2 infection, transient viral replication in the heart triggered macrophage-predominant immune infiltration and subsequent deterioration in cardiac function, supporting the concept that primary cardiac infection can be sufficient to drive myocardial dysfunction *in vivo* (41). These mechanistic observations, while informative, should be interpreted alongside human tissue data indicating substantial interindividual variability in the degree of myocardial viral burden and inflammatory injury.

Beyond the acute phase, a subset of patients experience persistent symptoms and organ dysfunction after SARS-CoV-2 infection (post-acute sequelae of COVID-19, often termed “long COVID”). Emerging evidence suggests that ongoing cardiovascular manifestations may occur in this context, including persistent myocardial inflammation and dysautonomia, although the underlying biology is not fully defined (42). In some studies, SARS-CoV-2 viral RNA and related viral material have been identified in cardiac tissues for months after the initial infection, with distribution across distinct cardiac regions, including myocardial tissue and coronary arteries. Such findings support the possibility that persistent viral material and/or sustained immune activation may contribute to post-COVID myocardial and vascular pathology in a subset of patients. (8, 37, 43). Importantly, population-based data from the United Kingdom indicate that the risk of acute myocarditis following SARS-CoV-2 vaccination is lower than the risk observed after SARS-CoV-2 infection in unvaccinated individuals, supporting an overall favorable risk–benefit profile of vaccination for myocarditis prevention at the population level (44). Overall, SARS-CoV-2 can affect the heart during acute infection and may also impose longer-term cardiovascular risk, either by inducing new pathology or by exacerbating pre-existing cardiovascular diseases. Accordingly, elucidating the mechanisms, incidence, and trajectory of cardiovascular sequelae after SARS-CoV-2 infection remains an important priority with direct implications for prevention, risk stratification, and long-term management.

### Influenza virus

Seasonal influenza remains a major cause of global morbidity and mortality, and the periodic emergence of novel strains with pandemic potential continues to pose a substantial public health threat (45). Beyond respiratory diseases, influenza infection can precipitate or exacerbate cardiovascular dysfunction, and a growing body of clinical and experimental evidence supports an association between influenza-related cardiovascular complications and adverse outcomes (11, 46, 47). Population-based studies further indicate that pre-existing cardiovascular diseases are associated with increased mortality during influenza infection, and that acute influenza illness is temporally linked to an increased risk of cardiovascular events (4, 47, 48).

The mechanisms by which influenza contributes to cardiac injury appear to be multifactorial. Both influenza A and B viruses have been implicated in cardiac complications, potentially through cytokine-mediated cardiotoxicity and immune dysregulation, including autoimmune responses directed against cardiac antigens (49–52). Cardiac injury may occur via direct infection of cardiac cells or indirectly through systemic inflammation with induction of inflammatory mediators, acute-phase reactants, and procoagulant factors, which together can promote endothelial dysfunction, microvascular injury, and thrombosis (46, 49, 53). In line with these mechanisms, epidemiologic studies have reported a significant association between recent influenza infection and acute myocardial infarction (54), supporting the concept that influenza can trigger plaque destabilization and/or a prothrombotic milieu leading to acute coronary syndromes. Experimental studies further support a direct role of influenza virus in cardiac pathology. Kenney and colleagues showed that influenza-infected Ifitm3^−/−^ mice developed significant cardiac electrical abnormalities, including bradycardia and irregular RR intervals, consistent with direct cardiac involvement. Importantly, by using a recombinant influenza virus that was specifically attenuated for replication in cardiomyocytes but retained replication competence in the lungs, they further demonstrated that pulmonary inflammation alone was not sufficient to drive the cardiac phenotype. Rather, direct cardiomyocyte infection and replication were required for the development of influenza-associated cardiac dysfunction and fibrosis in this model (46, 55, 56). Recent research has further elucidated a unique cellular route for influenza virus dissemination to the heart and its local pathogenic mechanisms within cardiac tissue. Work by Downey et al. revealed that the influenza virus can traffic to the heart via a specialized immune cell subset termed progenitor DC3 (pro-DC3). This study also established that, in the context of direct cardiomyocyte infection, type I interferon signaling within cardiomyocytes itself is cardiopathogenic, uncovering a novel intracellular mechanism by which viral infection precipitates cardiac injury (52). Complementary animal data indicate that both pathogenic and attenuated human influenza A viruses can infect multiple cardiac cell types—including cardiomyocytes and components of the specialized conduction system (e.g., Purkinje cells)—as well as cardiac endothelial cells, and that cardiac infection may occur independently of pulmonary viral titers, suggesting tissue-specific determinants of viral dissemination and tropism (7).

Collectively, available data support several non-mutually exclusive pathways for influenza-associated cardiac injury, including direct cardiomyocyte injury, systemic and interferon-driven inflammatory responses, excessive cytokine signaling, myocardial fibrosis and remodeling, hypoxia-related supply–demand mismatch, and destabilization of coronary atherosclerotic plaques (46, 52, 57–59). Importantly, influenza vaccination reduces the risk of infection and is associated with fewer influenza-related cardiovascular complications. By preventing infection and attenuating systemic inflammatory responses, vaccination may thereby reduce the incidence of influenza-associated myocarditis and heart failure exacerbations (60). Nevertheless, the relative contribution of each pathogenic pathway likely varies across patient populations and clinical phenotypes, and the mechanisms underlying influenza-associated cardiac events in humans are not fully resolved. Further mechanistic and translational studies are therefore needed to refine risk stratification and to inform personalized diagnostic and therapeutic strategies aimed at reducing the burden of influenza-related cardiovascular complications.

### HIV

HIV remains a major global public health challenge, with an estimated 44.1 million cumulative deaths to date. According to the latest World Health Organization report, approximately 40.8 million people were living with HIV by the end of 2024, with about 65% residing in the WHO African Region (61). HIV primarily infects and depletes CD4+ T lymphocytes, which are critical for host immune competence. In the absence of treatment, progressive CD4+ T-cell loss leads to severe immunodeficiency and, ultimately, acquired immunodeficiency syndrome (AIDS) (62). With improved access to and effectiveness of antiretroviral therapy (ART), HIV infection has transitioned to a chronic, manageable condition. Consequently, people living with HIV/AIDS increasingly experience non-AIDS comorbidities, including cardiovascular disease (CVD), often at a younger age than HIV-negative populations (63, 64). Cardiovascular and other circulatory system disorders have emerged as prominent causes of morbidity among people living with HIV/AIDS, driven by a multifactorial pathogenesis that includes persistent immune activation and chronic inflammation, HIV-associated endothelial dysfunction, and metabolic disturbances related in part to ART exposure (65, 66). Epidemiological and clinical studies indicate that CVD is now a leading contributor to HIV-associated morbidity and mortality, with a significantly elevated cardiovascular risk observed in people living with HIV/AIDS compared with the general population (67). Importantly, although ART can achieve durable suppression of plasma viremia, it does not eradicate latent viral reservoirs. Viral proteins may continue to contribute to pathogenesis even under virologic suppression. The HIV-1 accessory protein Nef has been implicated in viral virulence, immune dysregulation, and persistence. In Nef transgenic (Nef-TG) mouse models, Nef expression has been associated with impaired cardiac function, adverse myocardial remodeling, and increased fibrosis, culminating in heart failure and premature death. Mechanistically, Nef-mediated inhibition of autophagy and induction of aging-associated markers have been proposed to promote cellular senescence, potentially contributing to cardiovascular aging in people living with HIV/AIDS (68).

Chronic HIV-associated inflammation promotes endothelial dysfunction, accelerates atherogenesis, and increases the risk of ischemic events such as myocardial infarction and stroke. Elevated levels of inflammatory biomarkers, including C-reactive protein (CRP), have been associated with vascular injury and ongoing cardiovascular risk in HIV-infected individuals (69, 70). HIV-associated cardiomyopathy is likewise heterogeneous, and its etiology and clinical phenotype depend partly on the degree of immunodeficiency (71, 72). In individuals with uncontrolled viral replication and advanced immunosuppression, cardiomyopathy is more commonly linked to myocarditis due to direct or indirect viral injury, opportunistic infections, and nutritional deficiencies (73). In contrast, among ART-treated individuals with sustained viral suppression, contributory mechanisms more frequently include immune-mediated myocardial injury, persistent low-grade inflammation, and antiretroviral drug–related cardiotoxicity (74, 75). Despite substantial progress, the mechanisms linking HIV infection to heart disease remain incompletely defined. Further investigation is needed to disentangle the respective contributions of HIV-related immune activation, premature aging, traditional cardiovascular risk factors, and long-term ART exposure, as well as their interactions over the life course.

### CVB3

**Fig 2:**
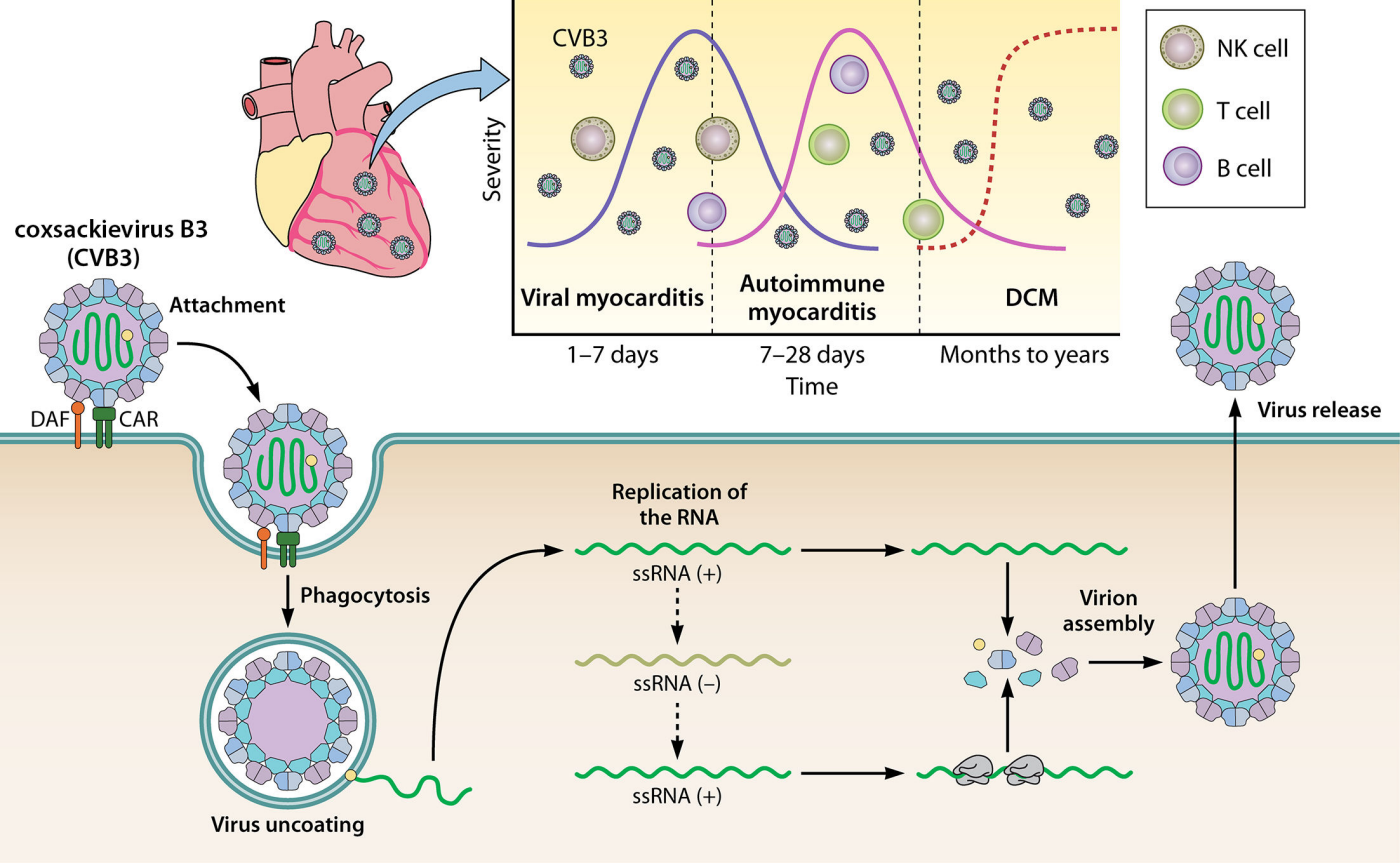
Mechanism of CVB3 infection leading to myocarditis. CVB3: coxsackieviruses B3; CAR: coxsackievirus-adenovirus receptor; DAF: decay-accelerating factor; DCM: dilated cardiomyopathy.

Enteroviruses are among the most frequently implicated pathogens in viral myocarditis, with group B coxsackieviruses (CVBs), particularly CVB3, historically linked to both acute myocarditis and subsequent progression to chronic inflammatory cardiomyopathy and dilated cardiomyopathy (76). The association between acute myocarditis and CVB infection was recognized as early as the mid-1950s, when multiple studies reported isolation of coxsackieviruses from myocardial or pericardial specimens in patients with acute myocarditis (12, 77). A central determinant of cardiac susceptibility to coxsackievirus infection is the coxsackievirus and adenovirus receptor (CAR), which mediates viral attachment and entry. Beyond its role in viral uptake, CAR participates in cytoskeletal organization and intracellular signaling, suggesting that CAR engagement and downstream perturbations may contribute to myocardial dysfunction during infection (78, 79). Infection of cardiomyocytes can trigger direct cytopathic injury and a robust inflammatory response, resulting in interstitial edema, necrosis, and impaired contractility. Clinically, patients may present with chest pain, palpitations, or dyspnea, and severe cases can progress to dilated cardiomyopathy and heart failure (80, 81) (Fig. 2). Experimental studies further support mechanistic links between CVB infection and contractile and electrical instability. In transgenic murine models expressing CVB3, investigators have observed reduced stroke volume and cardiac output, mild QTc prolongation, intracellular Ca²^+^ overload, contractile dysfunction, and mitochondrial dysregulation. These abnormalities disrupt excitation–contraction coupling and may increase susceptibility to stress-induced arrhythmias (82, 83).

Host immune responses are critical determinants of disease severity and outcome in CVB3 myocarditis, contributing to both viral control and immunopathology. Recent work has identified several potentially targetable pathways, including complement signaling (e.g., C4/C3 axis) with links to ferroptotic cell death in infected cardiomyocytes, macrophage-derived VEGF-C–mediated remodeling of cardiac lymphatics, metabolic reprogramming of neutrophils by α-lipoic acid, and attenuation of inflammation via blockade of IL1RAP, which has been reported to reduce disease severity in acute CVB3 myocarditis and experimental autoimmune myocarditis models (84–87). In addition, mitochondrial calpain-1 has been reported to suppress NLRP3 inflammasome activation via mitochondrial pathways, thereby mitigating inflammation and cardiac dysfunction in CVB3-induced viral myocarditis (88). Mitochondrial quality control, extracellular vesicle signaling, and noncoding RNAs also appear to modulate CVB3 pathogenesis. Parkin-dependent mitophagy has been implicated in regulating the development and progression of viral myocarditis. Exosomes derived from cardiac progenitor cells have been reported to inhibit CVB3 replication and modulate mTOR signaling with functional improvement in experimental viral myocarditis, whereas miR-19a/19b has been implicated in promoting viral biosynthesis and replication, thereby exacerbating myocardial injury (89–91). Nonpharmacologic approaches, such as low-intensity pulsed ultrasound, have also been reported to attenuate CVB3-induced cardiac inflammation in experimental settings (92).

Despite substantial mechanistic progress, clinical management remains largely supportive. Most translational and clinical research on enterovirus-related cardiovascular diseases has focused on myocarditis and its progression to inflammatory cardiomyopathy, whereas other potential cardiac manifestations are comparatively less well characterized. Moreover, there are currently no widely used, enterovirus-specific vaccines or approved antiviral therapies for CVB myocarditis, underscoring the need for effective preventive and disease-modifying strategies for enterovirus-associated myocardial disease.

### Human cytomegalovirus

HCMV is a globally prevalent β-herpesvirus that establishes lifelong infection. Following primary infection, HCMV is commonly asymptomatic in immunocompetent hosts and then persists in a latent state with intermittent reactivation. Infected individuals may shed virus for prolonged periods, thereby serving as a reservoir for transmission (93). Although HCMV typically causes limited clinical disease in immunocompetent individuals, it can lead to significant morbidity in immunocompromised patients, particularly in the setting of transplantation and other forms of iatrogenic or disease-related immunosuppression. Accumulating evidence links HCMV infection to cardiovascular pathology, including myocarditis, transplant vasculopathy, atherosclerosis/arteriosclerosis, and hypertension (13, 94–97). In immunosuppressed patients, viral reactivation is common and has been proposed to contribute to cardiovascular injury through sustained inflammatory and vascular effects (98). In addition, latency and intermittent reactivation may provide a mechanistic basis for chronic or recurrent inflammatory cardiac phenotypes, including myocarditis, although causality and the magnitude of effect likely vary by host factors and clinical context.

Epidemiologic data on HCMV and coronary heart disease (CHD) remain mixed. Meta-analyses of case–control studies suggest an association between HCMV seropositivity and increased CHD risk, whereas more recent cohort studies have not consistently reproduced this relationship, highlighting the potential impact of confounding, differences in outcome definitions, and heterogeneity in study populations (99, 100). Importantly, although uncommon, CMV-associated myocarditis and heart failure have been reported even in immunocompetent young adults, supporting the possibility of clinically significant cardiac involvement outside classic high-risk groups (101). Experimental models provide mechanistic support for CMV-associated cardiac injury. In a murine/rat cytomegalovirus (MCMV) infection model, infection has been shown to induce myocardial injury and functional impairment during both acute infection and latency. Viral G protein–coupled receptor homologs (vGPCRs), including US28 and M33, have been implicated in promoting long-term cardiac dysfunction (102, 103). In transplantation models, a latent CMV milieu may facilitate viral reactivation after heart transplantation, potentially influencing graft inflammation and survival. Moreover, MCMV infection induces pro-inflammatory cytokine production in BALB/c mice, and downregulation of miR-1929-3p has been linked to myocardial remodeling via endothelin A receptor–dependent activation of the NLRP3 inflammasome, affecting the endocardium, epicardium, and myocardium (104, 105).

Overall, HCMV is associated with a range of cardiovascular conditions, particularly in immunosuppressed hosts and transplant settings. However, the extent to which HCMV is a causal driver versus a modifier of cardiovascular risk remains incompletely defined, and the dominant pathogenic pathways in the human heart—spanning direct tissue infection, immune dysregulation, and vascular inflammation—require further clarification.

### Arthropod-borne viruses (arboviruses)

DENV, CHIKV, and ZIKV are prevalent arboviruses transmitted mainly by hematophagous arthropods (predominantly Aedes mosquitoes) and impose a disproportionate burden in low- and middle-income countries (106). Although these infections most commonly present as acute febrile illnesses, cardiovascular involvement is increasingly recognized and may contribute to severe diseases and mortality (107–109).

For DENV, pooled estimates suggest that cardiac events occur in a substantial proportion of cases (reported global incidence ~27.21%), and dengue-associated myocarditis appears more frequent in younger individuals, particularly those <20 years of age (110). Clinical and epidemiologic studies have linked DENV infection to myocardial injury and structural/functional changes, myocarditis, arrhythmias, heart failure, and shock (9, 107, 110). In addition, population-level analyses indicate that the risk of cardiovascular events—including major adverse cardiac events (MACE), arrhythmias, and ischemic cardiac diseases—may be increased during the early convalescent period, particularly within 30 days after DENV infection (107). Mechanistic work, including studies focused on DENV-3, implicates inflammatory infiltration, oxidative stress, and electrophysiologic remodeling as contributors to cardiac dysfunction. In BALB/c mice, DENV-3 infection has been reported to reduce cardiac output and stroke volume, decrease left ventricular dimensions/volumes at end-systole and end-diastole, and impair ventricular cardiomyocyte calcium currents, accompanied by leukocyte infiltration, myocardial inflammation, increased reactive oxygen species, and lipid peroxidation (111). In experimental settings, DENV-associated cardiac infection and injury have been observed mainly in interferon signaling-deficient or otherwise highly susceptible mouse models, in which viral presence in cardiac tissue is accompanied by inflammatory infiltration, myocardial fibrosis, and impaired cardiac function. While these findings indicate that DENV can involve the heart under permissive conditions, such models do not recapitulate productive infection in adult immunocompetent mice. Therefore, they should be interpreted primarily as evidence from susceptibility-enhanced systems rather than as definitive proof of how innate immunity or type I interferon responses govern DENV-associated cardiac injury in immunocompetent hosts (112). Despite these observations, mechanistic and translational studies remain relatively limited, and robust clinical predictors and targeted management strategies are not yet well established.

Chikungunya virus (CHIKV) infection can also involve the cardiovascular system (113). Human pathological studies have provided direct evidence of cardiac involvement, demonstrating the presence of CHIKV within cardiac tissue. In addition, CHIKV RNA has been detected in the heart as part of a broader pattern of multi-organ infection, accompanied by inflammatory cell infiltration in several affected organs, including the heart (114). These findings support the concept that direct viral invasion, together with associated inflammatory responses, may contribute to CHIKV-related cardiac pathology. Reported cardiovascular manifestations include hypotension, shock, circulatory failure, arrhythmias, myocarditis, dilated cardiomyopathy, and heart failure (14). A systematic synthesis of available studies estimated a global incidence of cardiac complications of ~32.81% (110). Furthermore, beyond the acute phase, CHIKV infection is associated with an increased risk of death from cerebrovascular disease, ischemic heart disease, and diabetes, indicating potential long-term cardiovascular sequelae (113). Elevated circulating cardiovascular biomarkers in fatal cases further support the presence of endothelial and/or myocardial injury (115). Experimental data indicate that CHIKV can directly infect cardiac tissue in immunocompetent mice and human primary cardiac cells, with cardiac fibroblasts representing a major target cell population. In immunocompetent hosts, cardiac infection is effectively controlled and cleared through a local type I interferon response. By contrast, loss of IFN-I signaling leads to increased cardiac viral burden, broader viral dissemination, and enhanced apoptosis within the heart (116, 117). In addition, MAVS signaling is required for efficient viral clearance from cardiac tissue; in its absence, persistent infection is associated with focal myocarditis and vasculitis of large vessels adjacent to the heart (118). These findings provide mechanistic insight into CHIKV–cardiac tissue interactions, although the precise pathways linking viral persistence to specific cardiovascular phenotypes remain incompletely defined.

ZIKV is best known for neurotropism and congenital diseases, but cardiovascular involvement has been described. In addition to typical symptoms (rash, headache, low-grade fever, arthralgia, myalgia, and conjunctivitis), some patients present with cardiovascular manifestations and may exhibit elevations in myocardial injury biomarkers (119). Case reports describe ZIKV-associated myocarditis and arrhythmias, including atrial fibrillation (10, 119, 120). *In vivo*, ZIKV-induced myocardial immune activation and myocarditis have been reported mainly in juvenile mice or in IFN-α/β receptor-deficient models, whereas adult wild-type mice generally show minimal permissiveness to infection (121). In adult A129 mice, ZIKV infection has been associated with electrocardiographic abnormalities, increased myocardial enzyme levels, downregulation of connexin 43 (Cx43), and disruption of gap junction architecture, findings consistent with acute myocardial injury and potential arrhythmogenic remodeling (122). However, these observations arise largely from susceptibility-enhanced models and should not be overinterpreted as direct evidence for the role of innate immunity or type I interferon pathways in immunocompetent adult hosts. More definitive conclusions regarding these mechanisms require appropriately supported data from mouse-adapted viral strains, humanized models, or human studies.

Taken together, ecological and societal drivers—including climate change, urbanization, and increased population mobility—are expanding the geographic range and outbreak potential of arboviruses, particularly in tropical and subtropical regions. Although cardiac involvement is an important and potentially life-threatening complication of DENV, CHIKV, and ZIKV infections, current evidence is heterogeneous, and mechanistic studies are relatively few. Further work is needed to clarify the incidence and risk factors for cardiovascular complications, define causal mechanisms (including the roles of direct viral tropism versus immune-mediated injury), and identify actionable targets for prevention, surveillance, and treatment.

## MECHANISMS OF VIRAL-INDUCED CARDIAC DISEASES

### Direct infection and indirect pathways

Many cardiotropic viruses can directly infect cardiomyocytes, resulting in cellular dysfunction and death (122). Following primary infection, typically in the respiratory or gastrointestinal tract, some viruses may disseminate through the bloodstream and/or lymphatic system and subsequently involve the heart. However, the route to cardiac injury and the underlying pathogenic mechanisms differ substantially among viruses. For example, coxsackievirus B, a prototypical cardiotropic enterovirus, can disseminate via viremia and directly infect cardiomyocytes (79). Influenza virus should likewise not be regarded as acting solely through indirect systemic effects, because experimental studies have demonstrated that direct infection and replication within cardiomyocytes are critical determinants of influenza-associated cardiac dysfunction, although systemic inflammation and immune activation may further exacerbate myocardial injury (46, 52). In contrast, HIV is not generally considered to productively infect cardiomyocytes, and its cardiovascular manifestations are thought to arise predominantly through indirect mechanisms, including chronic immune activation, persistent inflammation, endothelial dysfunction, and antiretroviral therapy-related toxicity. Therefore, once viruses reach cardiac tissue, some can directly enter and replicate within cardiomyocytes, whereas others contribute to myocardial injury mainly through host-mediated indirect pathways (6, 7, 68). Direct infection can injure cardiomyocytes through several non-mutually exclusive mechanisms. Viral replication and the accumulation of viral proteins may exert cytopathic effects that disrupt sarcomeric integrity, calcium handling, and electrical stability. In parallel, detection of viral nucleic acids and proteins by cardiomyocyte pattern-recognition receptors activates innate immune signaling cascades, inducing inflammatory mediators and stress responses that further impair contractile function. These intracellular events can converge on regulated cell-death pathways—including apoptosis, necroptosis, and other forms of lytic death—culminating in cardiomyocyte loss, reduced myocardial reserve, and clinically apparent myocardial dysfunction.

### Immune-mediated damage

In the pathogenesis of viral myocardial injury, dysregulated immune responses play a key role (123, 124), primarily involving two mechanisms. The first is molecular mimicry, wherein viral epitopes structurally resemble cardiac self-antigens, leading to cross-reactive immune responses that attack myocardial tissue (125, 126). CVB3 is a typical example; the antiviral antibodies it induces can cross-react with cardiomyocyte antigens, not only promoting the infiltration of immune cells such as T lymphocytes and macrophages but also—through an excessive humoral response—causing the deposition of immune complexes within myocardial tissue. This, in turn, activates the complement system and triggers immune complex-mediated inflammation, continuously disrupting myocardial structure and function (127). Additionally, excessive or sustained inflammatory responses represent another core mechanism. Acute infections can induce a “cytokine storm,” as seen in Zika virus infection, which triggers a cytokine release syndrome characterized by massive release of pro-inflammatory cytokines such as interleukin-6 and tumor necrosis factor-α, resulting in severe collateral tissue damage (128–130). Chronic infections, on the other hand, manifest as persistent immune activation. For example, in HIV infection, ongoing viral replication and immune dysregulation lead to CD4^+^ T-cell depletion, heightened T-cell activation, and sustained inflammatory signaling, which are considered central drivers of increased cardiovascular complication risk (131–133). These immune-mediated mechanisms, whether through autoimmune cross-reactivity or uncontrolled inflammatory responses, collectively contribute to myocarditis and may progress to cardiomyopathy.

### Effects of viruses on cardiac electrophysiology

**Fig 3:**
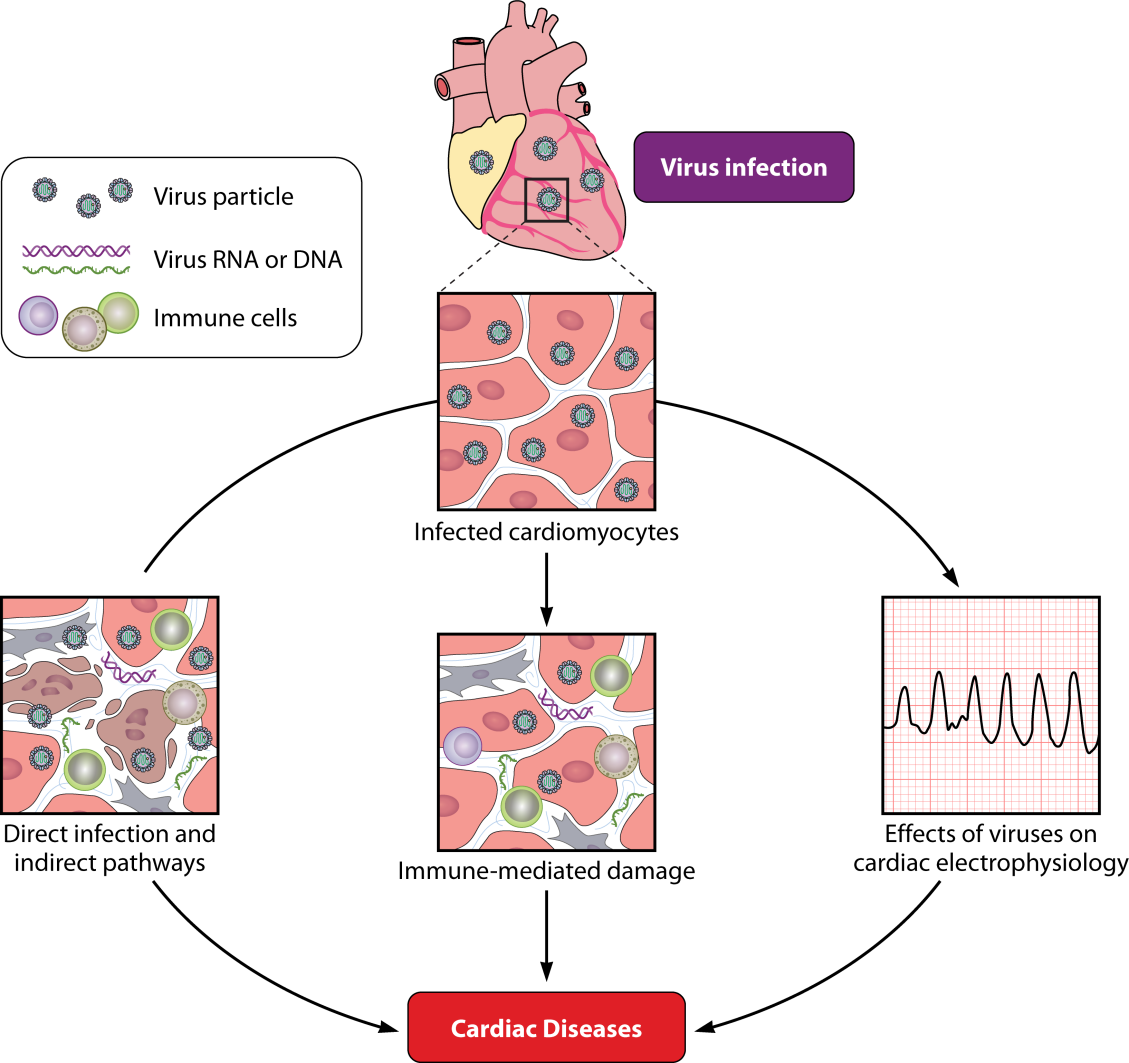
Mechanisms by which viruses trigger heart disease.

Viral infections can perturb cardiac electrophysiology and thereby promote arrhythmias through both direct effects on the cardiac conduction system and indirect, inflammation-mediated mechanisms. Coxsackievirus B3 (CVB3), for example, can infect cardiomyocytes via receptor-mediated entry involving the coxsackievirus and adenovirus receptor (CAR) and decay-accelerating factor (DAF/CD55). This may enable viral invasion of conduction tissue and disruption of normal impulse generation and propagation, predisposing to rhythm disturbances (134). Following SARS-CoV-2 infection, clinical observations have described sinus node dysfunction and atrioventricular (AV) conduction abnormalities, including sinus bradycardia and varying degrees of AV block (135). In HIV infection, viral proteins have been implicated in electrophysiological remodeling and may contribute directly to arrhythmogenic substrates in HIV-associated cardiac disease (136). Although the relative contributions of direct viral tropism versus secondary effects vary across patients, systemic inflammation, hypoxemia, autonomic imbalance, fever, electrolyte disturbances, and drug-related effects can all further modulate arrhythmic risk in the setting of acute infection. More broadly, immune-mediated myocardial injury and myocarditis can impair electrical coupling and conduction by damaging cardiomyocytes and the specialized conduction system and by altering the electrophysiological properties of surviving cells. Inflammatory cytokines, edema, microvascular dysfunction, and subsequent fibrosis can remodel ion channel expression and gap-junction integrity, thereby changing cardiomyocyte excitability, conduction velocity, and automaticity, and increasing susceptibility to both atrial and ventricular arrhythmias (137–141) (Table 1; Fig. 3).

**TABLE 1:** Cardiac manifestations caused by viral infection

Virus	Cardiac disease	Pathogenic mechanism
SARS-CoV-2	Myocarditis, arrhythmia, heart failure, myocardial infarction	Direct infection effects (142), secondary inflammatory responses (41), microcirculatory dysfunction (30)
Influenza virus	Myocardial infarction, arrhythmia, pericarditis, myocarditis, heart failure	Direct infection (49), infection transmission system (55), immune response (57)
HIV	Myocardial infarction, cardiomyopathy, myocarditis, heart failure	Direct toxicity (68), autoimmunity (69), nutritional deficiencies, and drugs (66)
CVB3	Myocarditis—dilated cardiomyopathy—heart failure	Direct toxicity (80), immune response (84), infection transmission system (83)
HCMV	Myocarditis, heart failure, infection after heart transplantation	latent infection (102), inflammasome-mediated myocardial remodeling (104)
Arboviruses (DENV, CHIKV, ZIKV)	Myocarditis, pericarditis, heart failure, arrhythmia, myocardial dysfunction, myocardial infarction	virulence (143), immune response (111), inflammatory pathways (118, 144), autophagy (145)

## TREATMENT STRATEGIES FOR VIRUS-RELATED CARDIAC DISEASES

### Antiviral therapy

For viral infection–associated heart disease, antiviral therapy is an important component of management when active viral replication is present or strongly suspected. Following myocardial infection, suppression of viral replication should be considered because reducing viral burden may attenuate downstream inflammatory responses and limit virus-mediated cardiomyocyte injury. Notably, the indication and expected benefit of antiviral therapy depend on the specific pathogen, the phase of disease (acute replicative vs. post-infectious immune-mediated), and host factors; therefore, treatment should be individualized and ideally guided by virologic and clinical evidence. Different viruses require distinct antiviral strategies. For enterovirus-associated myocarditis (e.g., Coxsackievirus B), ribavirin and other investigational approaches have been explored in experimental and limited clinical settings, although robust evidence and standardized regimens remain lacking in routine practice (146). In influenza-associated myocarditis or heart failure, neuraminidase inhibitors such as oseltamivir and zanamivir are recommended antiviral options, with the greatest benefit observed when initiated early after symptom onset (147–149). In SARS-CoV-2 infection, several antiviral agents have been evaluated, including entry inhibitors (e.g., chloroquine/hydroxychloroquine), protease inhibitors (e.g., lopinavir–ritonavir, darunavir), and RNA-dependent RNA polymerase inhibitors (e.g., remdesivir) (150–152); however, clinical efficacy varies by agent, and some drugs initially proposed (notably chloroquine/hydroxychloroquine and some HIV protease inhibitors) have not demonstrated clear benefit in well-controlled trials, emphasizing the need to align therapy with current evidence and guideline recommendations. In HIV infection, long-term combination ART is essential to achieve durable viral suppression and reduce systemic inflammation, thereby lowering the risk of cardiovascular complications over time. Across viral etiologies, earlier initiation of effective antiviral therapy—when indicated—generally confers greater benefit by limiting peak viral replication and mitigating subsequent inflammatory and immune-mediated injury.

### Immunomodulatory therapy

Immunomodulatory therapy is an important adjunct in selected forms of virus-related cardiac diseases, particularly when immune-mediated injury contributes substantially to myocardial dysfunction. Because antiviral immune responses can themselves drive cardiomyocyte damage, carefully modulating inflammation may reduce myocardial injury and improve clinical outcomes (153–155). Commonly discussed immunomodulatory approaches include glucocorticoids and intravenous immunoglobulin (IVIG), although the benefit of these interventions depends on patient selection, timing, and—critically—the presence or absence of ongoing myocardial viral replication. Glucocorticoids can suppress excessive inflammation and attenuate immune-mediated myocardial injury (154, 156). Accordingly, their use generally requires caution and is most defensible when active viral infection has been excluded or is considered unlikely. Prednisone has been reported to be beneficial in virus-negative inflammatory cardiomyopathy/myocarditis, supporting the concept that immunosuppression may be helpful in carefully phenotyped patients (150). In contrast, in patients with evidence of persistent myocardial viral infection, antiviral-oriented immunomodulation may be considered. The applicability of such an approach depends on the specific viral pathogen. For instance, in patients with persistent myocardial infection by enteroviruses or adenoviruses, therapy with interferon (IFN)-β has been associated with viral clearance and prevention of left ventricular functional deterioration. It is important to note, however, that the consequences of enhancing IFN signaling are context-dependent and may not be beneficial—and could potentially be harmful—in the setting of infections by other viruses (157). Beyond broad immunosuppression, more targeted immune modulation is under investigation. Experimental studies suggest that regulating T-cell trafficking can reduce CVB3-induced myocardial inflammation and fibrosis, thereby improving myocarditis severity and downstream remodeling (158, 159). These findings underscore the therapeutic potential of strategies that attenuate pathogenic immune pathways while preserving, or even enhancing, effective viral clearance. Overall, treatment development is challenged by the dual contribution of direct viral cytotoxicity and immune-mediated injury. Immunostimulatory approaches may accelerate viral elimination and could be advantageous early in infection, yet they risk amplifying inflammatory myocardial damage. Conversely, immunosuppressive therapies may relieve immune-mediated injury but can impair viral control and thereby worsen virus-driven pathology. Accordingly, a key unmet need is the development of biomarkers and diagnostic strategies (including myocardial virology, where feasible) that enable individualized selection and timing of immunomodulatory therapy. Immunomodulation is also a major area of interest in HIV-associated cardiac disease, where persistent immune activation and inflammation may contribute to endothelial dysfunction, myocardial injury, and adverse remodeling even under effective ART. Future work should define how best to calibrate immunomodulatory interventions in this setting—optimizing viral control while minimizing immune-mediated myocardial damage and avoiding impairment of host defenses—through mechanistic studies and rigorously designed clinical trials.

### Therapies to improve and support cardiac function

In patients with severe virus-related cardiac diseases—particularly those with acute myocarditis, decompensated heart failure, or malignant arrhythmias—hemodynamic stabilization and guideline-directed supportive care are central to management. Pharmacologic support may include diuretics to relieve congestion, vasodilators to reduce preload/afterload when blood pressure permits, and inotropic agents for patients with low cardiac output and evidence of end-organ hypoperfusion. These measures aim to optimize cardiac performance and reduce myocardial workload while the acute inflammatory and/or infectious process resolves. Arrhythmia management should be individualized according to the arrhythmia subtype, hemodynamic impact, and underlying myocardial involvement. In the context of SARS-CoV-2 infection, antiarrhythmic therapy (including class III agents in selected patients) may be used when clinically indicated, with careful attention to proarrhythmic risk, drug–drug interactions, and QT-interval monitoring (160). For patients at high risk of sudden cardiac death or with sustained ventricular tachyarrhythmias, device therapy such as an implantable cardioverter-defibrillator (ICD) may be considered in accordance with established indications and timing recommendations (often after reassessment of ventricular function once acute myocarditis has stabilized) (161). For patients with heart failure, treatment should follow contemporary heart failure guidelines, including beta-blockers and renin–angiotensin system inhibition (ACE inhibitors or angiotensin II receptor blockers, and, where appropriate, other guideline-directed agents), alongside diuretics for volume management. In refractory cases with progressive respiratory or circulatory failure, advanced organ support may be required. This can include invasive mechanical ventilation for severe hypoxemia and temporary mechanical circulatory support—such as extracorporeal membrane oxygenation (ECMO) in selected patients with cardiogenic shock—to maintain perfusion and oxygenation while allowing time for myocardial recovery and definitive etiologic therapy (162). For advanced, persistent heart failure despite optimal medical therapy, durable mechanical circulatory support such as left ventricular assist devices (LVADs) can be considered in carefully selected patients, including those with HIV infection when otherwise appropriate and consistent with transplant/LVAD candidacy criteria (163). Overall, the primary goal of cardiac support is to stabilize physiology, prevent secondary organ injury, and “bridge” patients through the acute phase to recovery or further interventions as needed.

### Treatment of long-term cardiovascular sequelae

Persistent or new symptoms occurring ≥12 weeks after acute SARS-CoV-2 infection are commonly referred to as long COVID (post-COVID-19 condition). Frequently reported cardiovascular-related complaints include fatigue, dyspnea, chest pain, and palpitations (164). Epidemiologic studies indicate that prior SARS-CoV-2 infection is associated with an increased risk of subsequent cardiovascular complications, including arrhythmias, myocarditis, pericarditis, ischemic cardiac disease, heart failure, and thromboembolic events (165). In parallel, post-infection evaluations have described abnormalities on cardiac magnetic resonance imaging (CMR) in some patients, as well as elevated cardiac biomarkers (e.g., troponin), persistent chest pain, and palpitations after apparent recovery; collectively, these findings support an increased incidence of cardiovascular events following SARS-CoV-2 infection, although the prevalence and clinical significance vary across cohorts and are influenced by baseline risk, infection severity, and ascertainment methods (163, 166). At present, management of cardiovascular manifestations in long COVID is largely individualized and symptom-directed, guided by standard cardiovascular diagnostic pathways to exclude alternative etiologies and to identify treatable conditions. For example, patients with chest pain suggestive of vasospastic or microvascular angina may benefit from anti-anginal therapy (e.g., calcium-channel blockers and/or nitrates) when clinically appropriate, while β-blockers (or alternatives such as ivabradine, depending on local practice and contraindications) may be considered for troublesome palpitations or inappropriate sinus tachycardia. Autonomic dysfunction, including postural orthostatic tachycardia syndrome (POTS), has been reported after SARS-CoV-2 infection. First-line therapy generally emphasizes non-pharmacologic measures (adequate hydration, increased salt intake when not contraindicated, compression garments, and graded recumbent exercise/physical reconditioning), with pharmacologic options reserved for persistent symptoms. Medications used in selected patients include volume-expanding agents (e.g., fludrocortisone), vasoconstrictors (e.g., midodrine), and heart-rate–lowering therapies (e.g., β-blockers), tailored to blood pressure profile and comorbidities. For patients with prominent fatigue syndromes, management typically focuses on pacing, sleep optimization, rehabilitation strategies, and treatment of comorbid mood, pain, or sleep disorders. Evidence for specific pharmacologic therapies (including immunomodulators or mitochondrial supplements such as coenzyme Q10) remains limited, and such interventions should be presented as investigational or adjunctive rather than as established standard of care. Similarly, in patients suspected of mast cell activation–related symptoms, H1/H2 antihistamines, mast cell stabilizers, and/or leukotriene receptor antagonists may be considered on a case-by-case basis. NSAIDs may be used for symptom relief (e.g., fever, myalgias, pleuritic pain), but they should be used cautiously in patients with heart failure, renal dysfunction, or elevated cardiovascular risk, consistent with general cardiovascular practice (35, 167).

## CONCLUSIONS AND FUTURE PERSPECTIVES

Over the past decade, the field of viral cardiology has undergone a profound paradigm shift. Historically centered on acute viral myocarditis—prototypically modeled by Enteroviruses such as Coxsackievirus B3—our understanding has evolved to recognize cardiotropic viruses as systemic drivers of a broad spectrum of major adverse cardiovascular events (MACEs), including acute myocardial infarction, heart failure, and chronic vascular dysfunction. This transition, significantly accelerated by the global COVID-19 pandemic, has been underpinned by technological advances such as single-cell sequencing, human pluripotent stem cell-derived cardiomyocytes (hPSC-CMs), and advanced cardiac magnetic resonance (CMR) imaging. Consequently, our mechanistic understanding has expanded to encompass novel viral trafficking routes (e.g., pro-DC3-mediated dissemination) and the complex, dual nature of immune responses, such as type I interferon signaling in cardiac protection versus injury.

Despite these unprecedented insights, critical knowledge gaps persist. It remains clinically challenging to disentangle direct viral cytopathic effects from indirect immune-mediated injuries, primarily due to the focal nature of infections and the limitations of endomyocardial biopsies. Furthermore, the mechanistic underpinnings of long-term cardiovascular sequelae—exemplified by "Long COVID" and premature cardiovascular aging in people living with HIV—remain poorly defined, particularly regarding the roles of persistent viral reservoirs and low-grade chronic inflammation in subclinical myocardial remodeling. Compounding these issues is a glaring lack of pathogen-specific antiviral and immunomodulatory therapies validated by large-scale clinical trials.

To address these challenges, future research must pivot toward precision medicine and long-term prevention strategies. First, the development of non-invasive, high-sensitivity biomarkers is urgently needed to distinguish active viral replication from post-viral autoimmunity, thereby guiding the optimal timing and modality of interventions. Parallel to this, therapeutic discovery should shift from broad-spectrum immunosuppression to targeting pathogenic inflammatory cascades—such as the NLRP3 inflammasome—without compromising the host’s innate antiviral capacity. Furthermore, establishing large-scale longitudinal cohorts integrating multi-omics data is essential for tracking post-infection cardiovascular trajectories and facilitating the early identification of individuals at high risk for chronic sequelae, such as heart failure or accelerated atherosclerosis. Ultimately, prioritizing vaccine development against emerging cardiotropic viruses, building on the established cardioprotective efficacy of existing vaccines, represents a fundamental strategy for reducing the global burden of cardiovascular disease.

In summary, mitigating the cardiovascular impact of viral infections requires transitioning from a reactive clinical approach to a proactive, preventative paradigm. By seamlessly integrating clinical epidemiology with advanced molecular biology, the field is well-positioned to develop highly effective strategies to improve cardiovascular outcomes in an increasingly interconnected and virus-exposed world.
